# Supplementary material for: Mamo decodes hierarchical temporal gradients into terminal neuronal fate
Source: eLife. 2019 Sep 23;8:e48056. doi: 10.7554/eLife.48056 (PMC6764822; doi:10.7554/eLife.48056)
Supplement: Source code 1. [file elife-48056-code1.zip › mamo_elife_code/mamo-isoforms-eLife.html]

mamo-isoforms-eLife


# import system/3rd party libraries¶

In [1]:

```
#
try:
    from importlib import reload # for python 3
except:
    pass
import logging
reload(logging)
logging.basicConfig(format='%(levelname)s:%(message)s', level=logging.INFO, datefmt='%I:%M:%S')
import os
import subprocess

%matplotlib inline


import numpy as N
import pandas as PD
from matplotlib import pylab as P


import matplotlib
matplotlib.rcParams['pdf.fonttype'] = 42
```

# import local code¶

In [2]:

```
import plotutils as PU
```

# code to draw isoforms¶

In [3]:

```
# gc: genomic coordinates (chr,st,ed)
# tc: pre-mRNA transcript coordinates (0=>N0)
# ec: exon coordinates (mature mRNA) (0=>N1)
# pc: protein coordinates (0=>N2)
import bisect
from matplotlib.collections import BrokenBarHCollection

class CoordConverter(object):
    """
                    TSS  exon1            intron          ATG(101050)                 exon3
    gc:    ------100101=========100201-------------101000======101200------------103102===103502------
    tc:    -----------0=========   100--------------- 899======  1099------------  3001===  3401------
    tc2:   -----------0=========   100--------------- 139======   339------------   434===   834------
    ec:    -----------0=========   100                100======   200               200===   600------
    pc:    ------------------------------------------------0***50--------------------50***   183------
    ic:    -----------0=========1-----------------------2======3----------------------4===5-----------
    
    gc: genome coordinates
    tc: transcript coordinates
    tc2: transcript coordinates intron compressed
    ec: exon coordinates
    pc: protein coordinates
    ic: index coordinates
    
    """
    
    def __init__(self, exonsgtf, atgpos, stoppos, tc2factor=20, tc2min=100):
        self.exonsgtf = exonsgtf
        self.atg_gc = atgpos # in gc
        self.stop_gc = stoppos # in gc
        self.tc2factor = tc2factor
        self.strand = strand = exonsgtf['strand'].iloc[0]
        # gc <=> tc
        self.tst = tst = exonsgtf['st'].min()-1 # in gc
        self.ted = ted = exonsgtf['ed'].max() # in gc
        # exons in tc
        if strand == '+':
            self.atg_tc = atgpos - tst # in tc
            self.stop_tc = stoppos - tst # in tc
            self.ests = [(x-1)-tst for x in exonsgtf['st']]  # in tc
            self.eeds = [x-tst for x in exonsgtf['ed']]  # in tc
        else:
            self.atg_tc = ted - atgpos # in tc
            self.stop_tc = ted - stoppos # in tc
            self.ests = [ted-x for x in exonsgtf['ed']] # in tc
            self.eeds = [ted-(x-1) for x in exonsgtf['st']] # in tc
        self.eposs = sorted(self.ests+self.eeds) # in tc
        self.esizes = [y-x for x,y in zip(self.ests, self.eeds)] # exon sizes
        self.isizes = [y-x for x,y in zip(self.eeds[:-1], self.ests[1:])] # intron sizes
        self.isizes2 = [max(tc2min,N.ceil(x/tc2factor)) for x in self.isizes] # intron sizes compressed
        self.ests2 = [0] + list(N.cumsum([x+y for x,y in zip(self.esizes[:-1], self.isizes2)]))
        self.eeds2 = [self.esizes[0]] + list(self.esizes[0]+N.cumsum([x+y for x,y in zip(self.isizes2,self.esizes[1:])]))
        self.eposs2 = sorted(self.ests2+self.eeds2)
        self.escum = N.cumsum(self.esizes) # cumulative exon sizes

        self.atg_eidx = bisect.bisect_left(self.eposs, self.atg_tc) # atg exon number
        self.atg_esti = ai = bisect.bisect_left(self.ests, self.atg_tc)-1 # atg exon number
        self.atg_ec = self.tc2ec(self.atg_tc) # atg ec
        #print(self.atg_tc, ai, self.atg_eidx, self.ests, self.atg_ec, self.stop_tc)
        # 87208 4 7 [0, 2757, 20758, 87188, 103331, 103606, 105467] 1972 105767
        assert(self.atg_ec>=0)
        self.atg_ecpos = self.atg_tc - self.ests[ai] # where in the exon ATG starts
        self.stop_eidx = bisect.bisect_left(self.eposs, self.stop_tc) # stop exon number
        self.stop_esti = si = bisect.bisect_left(self.ests, self.stop_tc)-1 # stop exon number
        self.stop_ec = self.tc2ec(self.stop_tc) # stop ec
        #print(self.stop_tc, si, self.stop_eidx, self.ests, self.stop_ec)
        # 105767 6 13 [0, 2757, 20758, 87188, 103331, 103606, 105467] 4372
        self.stop_ecpos = self.stop_tc - self.ests[si] # where in the exon STOP is
        self.atg_tc2 = self.ests2[ai] + self.atg_ecpos
        self.stop_tc2 = self.ests2[si] + self.stop_ecpos
        
        # UTR, coding in tc
        s1,e1,s2,e2,s3,e3 = self.chop2(self.ests, self.eeds, self.atg_tc, self.stop_tc)
        self.utr5sts = s1 #self.ests[:ai+1]
        self.utr5eds = e1 #self.eeds[:ai] + [self.atg_tc]
        self.codingsts = s2 #[self.atg_tc] + self.ests[ai+2:si+1]
        self.codingeds = e2 #self.eeds[ai+1:si] + [self.stop_tc]
        self.utr3sts = s3 #[self.stop_tc] + self.ests[si+1:]
        self.utr3eds = e3 #self.eeds[si:]
        self.utrsts = self.utr5sts + self.utr3sts
        self.utreds = self.utr5eds + self.utr3eds
        
        # UTR, coding in tc2
        s1,e1,s2,e2,s3,e3 = self.chop2(self.ests2, self.eeds2, self.atg_tc2, self.stop_tc2)
        self.utr5sts2 = s1 #self.ests2[:ai+1]
        self.utr5eds2 = e1 #self.eeds2[:ai] + [self.atg_tc2]
        self.codingsts2 = s2 #[self.atg_tc2] + self.ests2[ai+2:si+1]
        self.codingeds2 = e2 #self.eeds2[ai+1:si] + [self.stop_tc2]
        self.utr3sts2 = s3 #[self.stop_tc2] + self.ests2[si+1:]
        self.utr3eds2 = e3 #self.eeds2[si:]
        self.utrsts2 = self.utr5sts2 + self.utr3sts2
        self.utreds2 = self.utr5eds2 + self.utr3eds2
        
    def chop(self, sts, eds, pos):
        # chop into two sets at pos
        i = len(sts)
        # exon?
        for i in range(i):
            if (sts[i]<=pos) and (pos<=eds[i]): # in exon
                sts1 = sts[:i+1]
                eds1 = eds[:i] + [pos]
                sts2 = [pos] + sts[i+1:]
                eds2 = eds[i:]
                return [list(x) for x in [sts1,eds1,sts2,eds2]]
        # intron?
        for i in range(i-1):
            if (eds[i]<pos) and (pos<sts[i+1]): # in intron
                sts1 = sts[:i+1]
                eds1 = eds[:i+1]
                sts2 = sts[i+1:]
                eds2 = eds[i+1:]
                return [list(x) for x in [sts1,eds1,sts2,eds2]]
        if pos<sts[0]:
            return [],[],list(sts),list(eds)
        return list(sts),list(eds),[],[]
        
    def chop2(self, sts, eds, pos1, pos2):
        assert(pos1<pos2)
        s1,e1,s2,e2 = self.chop(sts, eds, pos1)
        s3,e3,s4,e4 = self.chop(s2, e2, pos2)
        return s1,e1,s3,e3,s4,e4
        
    def gc2tc(self, gpos):
        if self.strand=='+':
            return gpos - self.tst
        return self.ted - gpos
        
    def gc2tc2(self,gpos):
        tc = self.gc2tc(gpos)
        ec = self.tc2ec(tc)
        tc2 = self.ec2tc2(ec)
        return tc2
        
    def gc2pc(self, gpos):
        tc = self.gc2tc(gpos)
        ec = self.tc2ec(tc)
        pc = self.ec2pc(ec)
        return pc
        
    def tc2gc(self, tpos):
        if self.strand=='+':
            return self.tst + tpos
        return self.ted - tpos
        
    def tc2ec(self, tpos):
        # remove introns
        eidx = bisect.bisect_left(self.eposs, tpos)
        if eidx % 2 == 0: # intron
            return -1
        enum = (eidx-1)/2 # exon number
        # eidx-1(start) => eidx(end)
        if enum==0: # within exon1
            return tpos
        ec = self.escum[int(enum-1)]+(tpos - self.eposs[int(eidx-1)])
        return ec
        
    def epos2enum(self, epos):
        return bisect.bisect_left(self.escum, epos)
        
    def ec2tc(self, epos):
        eidx = self.epos2enum(epos)
        if eidx==0: # first exon
            d = epos # bp from exon start
        else:
            d = epos - self.escum[eidx-1] # bp from exon start
        tc = self.ests[eidx] + d
        return tc
        
    def ec2tc2(self, epos):
        eidx = self.epos2enum(epos)
        if eidx==0:
            d = epos
        else:
            d = epos - self.escum[eidx-1]
        tc2 = self.ests2[eidx] + d
        return tc2
        
    def ec2pc(self, epos):
        return int(N.ceil((epos-self.atg_ec) / 3.))
        
    def pc2ec(self, ppos):
        return self.atg_ec + 3*ppos
        
    def pc2gc(self, ppos):
        return self.tc2gc(self.ec2tc(self.pc2ec(ppos)))
        
    def eint2gint(self, est, eed):
        gst = self.tc2gc(self.ec2tc(est))
        ged = self.tc2gc(self.ec2tc(eed))
        if self.strand=='+':
            return (gst,ged)
        return (ged,gst)
        
    def eint2tint(self, est, eed):
        tst = self.ec2tc(est)
        ted = self.ec2tc(eed)
        return (tst, ted)
        
    def eint2tint2(self, est, eed):
        tst = self.ec2tc2(est)
        ted = self.ec2tc2(eed)
        return (tst,ted)
        
    def eint2iint(self, est, eed):
        ist = self.tc2ic(self.ec2tc(est))
        ied = self.tc2ic(self.ec2tc(eed))
        return (ist,ied)
        
    def pint2eints(self, pst, ped):
        ecst = self.pc2ec(pst) # start in ec
        eced = self.pc2ec(ped) # end in ec
        enst = self.epos2enum(ecst) # start exon number
        ened = self.epos2enum(eced) # end exon number
        if enst == ened:
            return [(ecst,eced)]
        escum = self.escum
        eints = [(ecst,escum[enst])]
        for i in range(enst+1,ened):
            eints.append((escum[i-1]+1,escum[i]))
        eints.append((escum[ened-1]+1,eced))
        return eints
            
    def pint2gints(self, pst, ped):
        eints = self.pint2eints(pst, ped)
        gints = [self.eint2gint(*x) for x in eints]
        return gints
        
    def pint2tints(self, pst, ped):
        eints = self.pint2eints(pst, ped)
        tints = [self.eint2tint(*x) for x in eints]
        return tints
            
    def pint2tints2(self, pst, ped):
        eints = self.pint2eints(pst, ped)
        tints = [self.eint2tint2(*x) for x in eints]
        return tints
        
    def tc2ic(self, tpos):
        eposs = self.eposs
        idx = bisect.bisect_left(eposs, tpos)-1
        isize = eposs[idx+1]-eposs[idx]
        frac = float(tpos-eposs[idx])/isize
        ic = idx+frac
        return ic
        
    def pint2iints(self, pst, ped):
        eints = self.pint2eints(pst, ped)
        iints = [self.eint2iint(*x) for x in eints]
        return iints
        
    def draw_exons_ic(self, ax, color='gray', alpha=0.7, ymid=0, h=0.8, ls='-'):
        x0 = 0
        x1 = len(self.eposs)-1  
        cargs = dict(facecolor=color, edgecolor=color, linewidth=0, alpha=alpha)
        ax.plot([x0,x1],[ymid,ymid],ls=ls, color=color)
        yrange = (ymid-h/2., h)
        xranges = [(x,1) for x in range(0,x1,2)]
        bbhc = BrokenBarHCollection(xranges, yrange, **cargs)
        ax.add_collection(bbhc)  
        
    def draw_pint_ic(self, ax, pst, ped, color='r', alpha=0.7, ymid=0, h=0.8, ls='-'):
        iints = self.pint2iints(pst, ped)
        x0 = iints[0][0]
        x1 = iints[-1][1]
        cargs = dict(facecolor=color, edgecolor=color, linewidth=0, alpha=alpha)
        ax.plot([x0,x1],[ymid,ymid],ls=ls, color=color)
        yrange = (ymid-h/2., h)
        xranges = [(x,y-x) for x,y in iints]
        bbhc = BrokenBarHCollection(xranges, yrange, **cargs)
        ax.add_collection(bbhc)  
        
    def draw_exons_tc(self, ax, color='gray', alpha=0.7, ymid=0, h_utr=0.6, h_coding=1, ls='-'):
        x0 = 0
        x1 = self.eposs[-1]
        cargs_utr = dict(facecolor=color, edgecolor=color, linewidth=0, alpha=alpha)
        cargs_coding = dict(facecolor=color, edgecolor=color, linewidth=0, alpha=alpha)
        yrange_utr = (ymid-h_utr/2., h_utr)
        yrange_coding = (ymid-h_coding/2., h_coding)
        ax.plot([x0+10,x1-10],[ymid,ymid],ls=ls, color=color, alpha=0.3)
        xranges_utr = [(x,y-x) for x,y in zip(self.utrsts,self.utreds)]
        xranges_coding = [(x,y-x) for x,y in zip(self.codingsts,self.codingeds)]
        bbhc_utr = BrokenBarHCollection(xranges_utr, yrange_utr, **cargs_utr)
        bbhc_coding = BrokenBarHCollection(xranges_coding, yrange_coding, **cargs_coding)
        ax.add_collection(bbhc_utr)
        ax.add_collection(bbhc_coding)
        
    def draw_pint_tc(self, ax, pst, ped, color='r', alpha=0.7, ymid=0, h_utr=0.6, h_coding=1, ls='-'):
        tints = self.pint2tints(pst, ped)
        sts,eds = zip(*tints)
        s1,e1,s2,e2,s3,e3 = self.chop2(sts,eds,self.atg_tc,self.stop_tc)
        x0 = tints[0][0]
        x1 = tints[-1][1]
        cargs = dict(facecolor=color, edgecolor=color, linewidth=0, alpha=alpha)
        ax.plot([x0+10,x1-10],[ymid,ymid],ls=ls, color=color)
        yrange_utr = (ymid-h_utr/2., h_utr)
        yrange_coding = (ymid-h_coding/2., h_coding)
        s_utr = s1+s3
        e_utr = e1+e3
        xranges_utr = [(x,y-x) for x,y in zip(s_utr,e_utr)]
        xranges_coding = [(x,y-x) for x,y in zip(s2,e2)]
        bbhc_utr = BrokenBarHCollection(xranges_utr, yrange_utr, **cargs)
        bbhc_coding = BrokenBarHCollection(xranges_coding, yrange_coding, **cargs)
        ax.add_collection(bbhc_utr)  
        ax.add_collection(bbhc_coding)  
        
    def draw_exons_tc2(self, ax, color='gray', alpha=0.7, ymid=0, h_utr=0.6, h_coding=1, ls='-', g0=None):
        x0 = 0
        x1 = self.eposs2[-1]
        cargs_utr = dict(facecolor=color, edgecolor=color, linewidth=0, alpha=alpha)
        cargs_coding = dict(facecolor=color, edgecolor=color, linewidth=0, alpha=alpha)
        yrange_utr = (ymid-h_utr/2., h_utr)
        yrange_coding = (ymid-h_coding/2., h_coding)
        if g0 is None:
            ax.plot([x0+10,x1-10],[ymid,ymid],ls=ls, color=color,alpha=alpha) # mid line
            xranges_utr = [(x,y-x) for x,y in zip(self.utrsts2,self.utreds2)]
            xranges_coding = [(x,y-x) for x,y in zip(self.codingsts2,self.codingeds2)]
        else:
            xori = self.gc2tc2(g0) # make this 0
            ax.plot([x0-xori+10,x1-xori-10],[ymid,ymid],ls=ls, color=color,alpha=0.3) # mid line
            xranges_utr = [(x-xori,y-x) for x,y in zip(self.utrsts2,self.utreds2)]
            xranges_coding = [(x-xori,y-x) for x,y in zip(self.codingsts2,self.codingeds2)]            
        bbhc_utr = BrokenBarHCollection(xranges_utr, yrange_utr, **cargs_utr)
        bbhc_coding = BrokenBarHCollection(xranges_coding, yrange_coding, **cargs_coding)
        ax.add_collection(bbhc_utr)
        ax.add_collection(bbhc_coding)
        
    def draw_pint_tc2(self, ax, pst, ped, color='r', alpha=0.7, ymid=0, h_utr=0.6, h_coding=1, ls='-',g0=None):
        tints = self.pint2tints2(pst, ped) # exon intervals in tc2 
        sts,eds = zip(*tints)
        s1,e1,s2,e2,s3,e3 = self.chop2(sts,eds,self.atg_tc2,self.stop_tc2)
        x0 = min(s1+s2+s3)#tints[0][0]
        x1 = max(e1+e2+e3)#tints[-1][1]
        cargs = dict(facecolor=color, edgecolor=color, linewidth=0, alpha=alpha)
        yrange_utr = (ymid-h_utr/2., h_utr)
        yrange_coding = (ymid-h_coding/2., h_coding)
        s_utr = s1+s3
        e_utr = e1+e3
        if g0 is None:
            ax.plot([x0+10,x1-10],[ymid,ymid],ls=ls, color=color)
            xranges_utr = [(x,y-x) for x,y in zip(s_utr,e_utr)]
            xranges_coding = [(x,y-x) for x,y in zip(s2,e2)]
        else:
            xori = self.gc2tc2(g0) # make this 0
            ax.plot([x0-xori+10,x1-xori-10],[ymid,ymid],ls=ls, color=color,lw=1) # mid line
            xranges_utr = [(x-xori,y-x) for x,y in zip(s_utr,e_utr)]
            xranges_coding = [(x-xori,y-x) for x,y in zip(s2,e2)] 
            #print(xranges_utr, xranges_coding, x0-xori, x1-xori)
        if len(xranges_utr)>0:
            bbhc_utr = BrokenBarHCollection(xranges_utr, yrange_utr, **cargs)
            ax.add_collection(bbhc_utr)
        bbhc_coding = BrokenBarHCollection(xranges_coding, yrange_coding, **cargs)
        bbhc_coding.set_zorder(1)
        ax.add_collection(bbhc_coding)  
                
    
def _drawone_tc2(ax, cc, g0, pints, label, colors):
    P.setp(ax, frame_on=False, xticks=[], yticks=[])
    ax.set_ylim(-0.6,0.6)
    x0 = cc.gc2tc2(g0)
    print(label, x0)#, cc.ests2, cc.eeds2)
    #ax.set_xlim(xlim)
    ax.set_ylabel(label, rotation=0, va='center')
    cc.draw_exons_tc2(ax=ax, g0=g0, alpha=0.7)
    for x,c in zip(pints,colors):
        cc.draw_pint_tc2(ax, *x, color=c, g0=g0, alpha=1.0)

def _drawone_tc(ax, cc, gint, pints, label, colors):
    P.setp(ax, frame_on=False, xticks=[], yticks=[])
    ax.set_ylim(-0.6,0.6)
    xlim = (cc.gc2tc(gint[0]), cc.gc2tc(gint[1]))
    print(xlim, cc.eposs[0], cc.eposs[-1])
    ax.set_xlim(xlim)
    if label is not None:
        ax.set_ylabel(label, rotation=0, va='center')
    cc.draw_exons_tc(ax=ax, alpha=0.7)
    for x,c in zip(pints,colors):
        cc.draw_pint_tc(ax, *x, color=c, alpha=1.0)
        
def _drawscalebar(ax, cc, gint, offset, size):
    P.setp(ax, frame_on=False, xticks=[], yticks=[])
    ax.set_ylim(-0.6,0.6)
    xlim = (cc.gc2tc(gint[0]), cc.gc2tc(gint[1]))
    print(xlim, cc.eposs[0], cc.eposs[-1])
    ax.set_xlim(xlim)
    ax.plot([xlim[0]+offset, xlim[0]+offset+size], [0,0], '-', lw=3)
```

# mamo structure info¶

In [4]:

```
mamo = PD.read_csv("mamo.csv", header=0, index_col=0) # extracted from dmel-all-r6.17.gtf 
mamo
```

Out[4]:

|  | chr | src | typ | st | ed | sc1 | strand | sc2 | extra | gene\_id | transcript\_id | gene\_name | transcript\_symbol |
| --- | --- | --- | --- | --- | --- | --- | --- | --- | --- | --- | --- | --- | --- |
| 432601 | X | FlyBase | exon | 13986371 | 13986442 | . | - | . | gene\_id "FBgn0267033"; gene\_symbol "mamo"; tra... | FBgn0267033 | FBtr0346001 | mamo | mamo-RI |
| 432602 | X | FlyBase | exon | 13985214 | 13986074 | . | - | . | gene\_id "FBgn0267033"; gene\_symbol "mamo"; tra... | FBgn0267033 | FBtr0346001 | mamo | mamo-RI |
| 432603 | X | FlyBase | exon | 13967270 | 13968073 | . | - | . | gene\_id "FBgn0267033"; gene\_symbol "mamo"; tra... | FBgn0267033 | FBtr0346001 | mamo | mamo-RI |
| 432604 | X | FlyBase | exon | 13901483 | 13901643 | . | - | . | gene\_id "FBgn0267033"; gene\_symbol "mamo"; tra... | FBgn0267033 | FBtr0346001 | mamo | mamo-RI |
| 432605 | X | FlyBase | exon | 13885309 | 13885500 | . | - | . | gene\_id "FBgn0267033"; gene\_symbol "mamo"; tra... | FBgn0267033 | FBtr0346001 | mamo | mamo-RI |
| 432606 | X | FlyBase | exon | 13883459 | 13885225 | . | - | . | gene\_id "FBgn0267033"; gene\_symbol "mamo"; tra... | FBgn0267033 | FBtr0346001 | mamo | mamo-RI |
| 432607 | X | FlyBase | exon | 13883236 | 13883364 | . | - | . | gene\_id "FBgn0267033"; gene\_symbol "mamo"; tra... | FBgn0267033 | FBtr0346001 | mamo | mamo-RI |
| 432608 | X | FlyBase | exon | 13856982 | 13857148 | . | - | . | gene\_id "FBgn0267033"; gene\_symbol "mamo"; tra... | FBgn0267033 | FBtr0346001 | mamo | mamo-RI |
| 432609 | X | FlyBase | exon | 13855235 | 13856759 | . | - | . | gene\_id "FBgn0267033"; gene\_symbol "mamo"; tra... | FBgn0267033 | FBtr0346001 | mamo | mamo-RI |
| 432610 | X | FlyBase | exon | 13854652 | 13855118 | . | - | . | gene\_id "FBgn0267033"; gene\_symbol "mamo"; tra... | FBgn0267033 | FBtr0346001 | mamo | mamo-RI |
| 432611 | X | FlyBase | exon | 13846116 | 13854372 | . | - | . | gene\_id "FBgn0267033"; gene\_symbol "mamo"; tra... | FBgn0267033 | FBtr0346001 | mamo | mamo-RI |
| 432628 | X | FlyBase | exon | 13988545 | 13988831 | . | - | . | gene\_id "FBgn0267033"; gene\_symbol "mamo"; tra... | FBgn0267033 | FBtr0346002 | mamo | mamo-RH |
| 432629 | X | FlyBase | exon | 13985214 | 13986074 | . | - | . | gene\_id "FBgn0267033"; gene\_symbol "mamo"; tra... | FBgn0267033 | FBtr0346002 | mamo | mamo-RH |
| 432630 | X | FlyBase | exon | 13967270 | 13968073 | . | - | . | gene\_id "FBgn0267033"; gene\_symbol "mamo"; tra... | FBgn0267033 | FBtr0346002 | mamo | mamo-RH |
| 432631 | X | FlyBase | exon | 13901483 | 13901643 | . | - | . | gene\_id "FBgn0267033"; gene\_symbol "mamo"; tra... | FBgn0267033 | FBtr0346002 | mamo | mamo-RH |
| 432632 | X | FlyBase | exon | 13885309 | 13885500 | . | - | . | gene\_id "FBgn0267033"; gene\_symbol "mamo"; tra... | FBgn0267033 | FBtr0346002 | mamo | mamo-RH |
| 432633 | X | FlyBase | exon | 13883459 | 13885225 | . | - | . | gene\_id "FBgn0267033"; gene\_symbol "mamo"; tra... | FBgn0267033 | FBtr0346002 | mamo | mamo-RH |
| 432634 | X | FlyBase | exon | 13883236 | 13883364 | . | - | . | gene\_id "FBgn0267033"; gene\_symbol "mamo"; tra... | FBgn0267033 | FBtr0346002 | mamo | mamo-RH |
| 432635 | X | FlyBase | exon | 13856982 | 13857148 | . | - | . | gene\_id "FBgn0267033"; gene\_symbol "mamo"; tra... | FBgn0267033 | FBtr0346002 | mamo | mamo-RH |
| 432636 | X | FlyBase | exon | 13855235 | 13856759 | . | - | . | gene\_id "FBgn0267033"; gene\_symbol "mamo"; tra... | FBgn0267033 | FBtr0346002 | mamo | mamo-RH |
| 432637 | X | FlyBase | exon | 13854652 | 13855118 | . | - | . | gene\_id "FBgn0267033"; gene\_symbol "mamo"; tra... | FBgn0267033 | FBtr0346002 | mamo | mamo-RH |
| 432638 | X | FlyBase | exon | 13850848 | 13854372 | . | - | . | gene\_id "FBgn0267033"; gene\_symbol "mamo"; tra... | FBgn0267033 | FBtr0346002 | mamo | mamo-RH |
| 432653 | X | FlyBase | exon | 13932482 | 13933343 | . | - | . | gene\_id "FBgn0267033"; gene\_symbol "mamo"; tra... | FBgn0267033 | FBtr0346003 | mamo | mamo-RE |
| 432654 | X | FlyBase | exon | 13901483 | 13901643 | . | - | . | gene\_id "FBgn0267033"; gene\_symbol "mamo"; tra... | FBgn0267033 | FBtr0346003 | mamo | mamo-RE |
| 432655 | X | FlyBase | exon | 13885309 | 13885500 | . | - | . | gene\_id "FBgn0267033"; gene\_symbol "mamo"; tra... | FBgn0267033 | FBtr0346003 | mamo | mamo-RE |
| 432656 | X | FlyBase | exon | 13883459 | 13885225 | . | - | . | gene\_id "FBgn0267033"; gene\_symbol "mamo"; tra... | FBgn0267033 | FBtr0346003 | mamo | mamo-RE |
| 432657 | X | FlyBase | exon | 13883236 | 13883364 | . | - | . | gene\_id "FBgn0267033"; gene\_symbol "mamo"; tra... | FBgn0267033 | FBtr0346003 | mamo | mamo-RE |
| 432658 | X | FlyBase | exon | 13870470 | 13871268 | . | - | . | gene\_id "FBgn0267033"; gene\_symbol "mamo"; tra... | FBgn0267033 | FBtr0346003 | mamo | mamo-RE |
| 432659 | X | FlyBase | exon | 13870320 | 13870403 | . | - | . | gene\_id "FBgn0267033"; gene\_symbol "mamo"; tra... | FBgn0267033 | FBtr0346003 | mamo | mamo-RE |
| 432660 | X | FlyBase | exon | 13858017 | 13869705 | . | - | . | gene\_id "FBgn0267033"; gene\_symbol "mamo"; tra... | FBgn0267033 | FBtr0346003 | mamo | mamo-RE |
| ... | ... | ... | ... | ... | ... | ... | ... | ... | ... | ... | ... | ... | ... |
| 432680 | X | FlyBase | exon | 13885309 | 13885500 | . | - | . | gene\_id "FBgn0267033"; gene\_symbol "mamo"; tra... | FBgn0267033 | FBtr0346004 | mamo | mamo-RD |
| 432681 | X | FlyBase | exon | 13883459 | 13885225 | . | - | . | gene\_id "FBgn0267033"; gene\_symbol "mamo"; tra... | FBgn0267033 | FBtr0346004 | mamo | mamo-RD |
| 432682 | X | FlyBase | exon | 13883236 | 13883364 | . | - | . | gene\_id "FBgn0267033"; gene\_symbol "mamo"; tra... | FBgn0267033 | FBtr0346004 | mamo | mamo-RD |
| 432683 | X | FlyBase | exon | 13870470 | 13871268 | . | - | . | gene\_id "FBgn0267033"; gene\_symbol "mamo"; tra... | FBgn0267033 | FBtr0346004 | mamo | mamo-RD |
| 432684 | X | FlyBase | exon | 13870320 | 13870403 | . | - | . | gene\_id "FBgn0267033"; gene\_symbol "mamo"; tra... | FBgn0267033 | FBtr0346004 | mamo | mamo-RD |
| 432685 | X | FlyBase | exon | 13869216 | 13869705 | . | - | . | gene\_id "FBgn0267033"; gene\_symbol "mamo"; tra... | FBgn0267033 | FBtr0346004 | mamo | mamo-RD |
| 432700 | X | FlyBase | exon | 13946950 | 13947076 | . | - | . | gene\_id "FBgn0267033"; gene\_symbol "mamo"; tra... | FBgn0267033 | FBtr0346005 | mamo | mamo-RF |
| 432701 | X | FlyBase | exon | 13914630 | 13915349 | . | - | . | gene\_id "FBgn0267033"; gene\_symbol "mamo"; tra... | FBgn0267033 | FBtr0346005 | mamo | mamo-RF |
| 432702 | X | FlyBase | exon | 13901483 | 13901643 | . | - | . | gene\_id "FBgn0267033"; gene\_symbol "mamo"; tra... | FBgn0267033 | FBtr0346005 | mamo | mamo-RF |
| 432703 | X | FlyBase | exon | 13885309 | 13885500 | . | - | . | gene\_id "FBgn0267033"; gene\_symbol "mamo"; tra... | FBgn0267033 | FBtr0346005 | mamo | mamo-RF |
| 432704 | X | FlyBase | exon | 13883459 | 13885225 | . | - | . | gene\_id "FBgn0267033"; gene\_symbol "mamo"; tra... | FBgn0267033 | FBtr0346005 | mamo | mamo-RF |
| 432705 | X | FlyBase | exon | 13883236 | 13883364 | . | - | . | gene\_id "FBgn0267033"; gene\_symbol "mamo"; tra... | FBgn0267033 | FBtr0346005 | mamo | mamo-RF |
| 432706 | X | FlyBase | exon | 13870320 | 13871268 | . | - | . | gene\_id "FBgn0267033"; gene\_symbol "mamo"; tra... | FBgn0267033 | FBtr0346005 | mamo | mamo-RF |
| 432707 | X | FlyBase | exon | 13869216 | 13869705 | . | - | . | gene\_id "FBgn0267033"; gene\_symbol "mamo"; tra... | FBgn0267033 | FBtr0346005 | mamo | mamo-RF |
| 432722 | X | FlyBase | exon | 13990470 | 13990527 | . | - | . | gene\_id "FBgn0267033"; gene\_symbol "mamo"; tra... | FBgn0267033 | FBtr0346006 | mamo | mamo-RG |
| 432723 | X | FlyBase | exon | 13985214 | 13986074 | . | - | . | gene\_id "FBgn0267033"; gene\_symbol "mamo"; tra... | FBgn0267033 | FBtr0346006 | mamo | mamo-RG |
| 432724 | X | FlyBase | exon | 13967270 | 13968073 | . | - | . | gene\_id "FBgn0267033"; gene\_symbol "mamo"; tra... | FBgn0267033 | FBtr0346006 | mamo | mamo-RG |
| 432725 | X | FlyBase | exon | 13901483 | 13901643 | . | - | . | gene\_id "FBgn0267033"; gene\_symbol "mamo"; tra... | FBgn0267033 | FBtr0346006 | mamo | mamo-RG |
| 432726 | X | FlyBase | exon | 13885309 | 13885500 | . | - | . | gene\_id "FBgn0267033"; gene\_symbol "mamo"; tra... | FBgn0267033 | FBtr0346006 | mamo | mamo-RG |
| 432727 | X | FlyBase | exon | 13883459 | 13885225 | . | - | . | gene\_id "FBgn0267033"; gene\_symbol "mamo"; tra... | FBgn0267033 | FBtr0346006 | mamo | mamo-RG |
| 432728 | X | FlyBase | exon | 13883236 | 13883364 | . | - | . | gene\_id "FBgn0267033"; gene\_symbol "mamo"; tra... | FBgn0267033 | FBtr0346006 | mamo | mamo-RG |
| 432729 | X | FlyBase | exon | 13870320 | 13871268 | . | - | . | gene\_id "FBgn0267033"; gene\_symbol "mamo"; tra... | FBgn0267033 | FBtr0346006 | mamo | mamo-RG |
| 432730 | X | FlyBase | exon | 13865862 | 13869705 | . | - | . | gene\_id "FBgn0267033"; gene\_symbol "mamo"; tra... | FBgn0267033 | FBtr0346006 | mamo | mamo-RG |
| 432745 | X | FlyBase | exon | 13988545 | 13988831 | . | - | . | gene\_id "FBgn0267033"; gene\_symbol "mamo"; tra... | FBgn0267033 | FBtr0346007 | mamo | mamo-RC |
| 432746 | X | FlyBase | exon | 13985214 | 13986074 | . | - | . | gene\_id "FBgn0267033"; gene\_symbol "mamo"; tra... | FBgn0267033 | FBtr0346007 | mamo | mamo-RC |
| 432747 | X | FlyBase | exon | 13967270 | 13968073 | . | - | . | gene\_id "FBgn0267033"; gene\_symbol "mamo"; tra... | FBgn0267033 | FBtr0346007 | mamo | mamo-RC |
| 432748 | X | FlyBase | exon | 13901483 | 13901643 | . | - | . | gene\_id "FBgn0267033"; gene\_symbol "mamo"; tra... | FBgn0267033 | FBtr0346007 | mamo | mamo-RC |
| 432749 | X | FlyBase | exon | 13885309 | 13885500 | . | - | . | gene\_id "FBgn0267033"; gene\_symbol "mamo"; tra... | FBgn0267033 | FBtr0346007 | mamo | mamo-RC |
| 432750 | X | FlyBase | exon | 13883459 | 13885225 | . | - | . | gene\_id "FBgn0267033"; gene\_symbol "mamo"; tra... | FBgn0267033 | FBtr0346007 | mamo | mamo-RC |
| 432751 | X | FlyBase | exon | 13882854 | 13883364 | . | - | . | gene\_id "FBgn0267033"; gene\_symbol "mamo"; tra... | FBgn0267033 | FBtr0346007 | mamo | mamo-RC |

64 rows × 13 columns

In [5]:

```
# get mamo isoforms
mamoisos = ['RC','RD','RE','RF','RG','RH','RI']

mamodfs = {x:mamo[mamo['transcript_symbol']=='mamo-{}'.format(x)] for x in mamoisos}
atg_gc = {x:13901623 for x in mamoisos}
stop_RC = 13883067 - 3
stop_RD = 13869556 - 3
stop_RH = 13854101 - 3
stop_gc = {
    'RC':stop_RC,
    'RD':stop_RD,
    'RE':stop_RD,   
    'RF':stop_RD,
    'RG':stop_RD,
    'RH':stop_RH,
    'RI':stop_RH,
}
mamoccs = {x:CoordConverter(mamodfs[x], atg_gc[x], stop_gc[x]) for x in mamoisos}

mamoclrs = {
    'RC':['c','k'],
    'RD':['c','r','b','m','k'],
    'RE':['c','r','b','m','k'],
    'RF':['c','r','g','b','m','k'],
    'RG':['c','r','g','b','m','k'],
    'RH':['c']+PU.hls6[:3]+PU.hls6[4:]+['k'],
    'RI':['c']+PU.hls6[:3]+PU.hls6[4:]+['k'],
}
```

In [6]:

```
# https://www.uniprot.org/uniprot/Q9VY72
# RH,RI
# FT   DOMAIN       31     97       BTB (POZ). {ECO:0000259|PROSITE:PS50097}.
# FT   DOMAIN     1114   1142       C2H2-type. {ECO:0000259|PROSITE:PS50157}.
# FT   DOMAIN     1147   1174       C2H2-type. {ECO:0000259|PROSITE:PS50157}.
# FT   DOMAIN     1469   1496       C2H2-type. {ECO:0000259|PROSITE:PS50157}.
# FT   DOMAIN     1497   1524       C2H2-type. {ECO:0000259|PROSITE:PS50157}.
# FT   DOMAIN     1525   1552       C2H2-type. {ECO:0000259|PROSITE:PS50157}.
pints_RH = [(31,97),(1114,1142),(1147,1174),(1469,1496),(1497,1524),(1525,1552),(273,286)]


# https://www.uniprot.org/uniprot/M9NEG1
# 66, 28, 27, 27, 27, 27
# RF,RG
# FT   DOMAIN       31     97       BTB (POZ). {ECO:0000259|PROSITE:PS50097}.
# FT   DOMAIN      800    827       C2H2-type. {ECO:0000259|PROSITE:PS50157}.
# FT   DOMAIN     1008   1035       C2H2-type. {ECO:0000259|PROSITE:PS50157}.
# FT   DOMAIN     1036   1063       C2H2-type. {ECO:0000259|PROSITE:PS50157}.
# FT   DOMAIN     1064   1086       C2H2-type. {ECO:0000259|PROSITE:PS50157}.
pints_RG = [(31,97),(800,827),(1008,1035),(1036,1063),(1064,1086),(273,286)]

# https://www.uniprot.org/uniprot/M9PJM9
# 66, 27, 27, 27, 22
# RD,RE
# FT   DOMAIN       31     97       BTB (POZ). {ECO:0000259|PROSITE:PS50097}.
# FT   DOMAIN      800    827       C2H2-type. {ECO:0000259|PROSITE:PS50157}.
# FT   DOMAIN     1014   1041       C2H2-type. {ECO:0000259|PROSITE:PS50157}.
# FT   DOMAIN     1042   1064       C2H2-type. {ECO:0000259|PROSITE:PS50157}.
pints_RD = [(31,97),(800,827),(1014,1041),(1042,1064),(273,286)]
# 66, 27, 27, 22

# https://www.uniprot.org/uniprot/H1UUK0
pints_RC = [(31,97),(273,286)]
pintsdic = {
    'RC':pints_RC,
    'RH':pints_RH,
    'RI':pints_RH,
    'RF':pints_RG,
    'RG':pints_RG,
    'RD':pints_RD,
    'RE':pints_RD
}
```

# draw mamo isoforms¶

In [7]:

```
DIR = '../'
```

In [8]:

```
fig, axr = P.subplots(7,1,figsize=(15,7*0.4),sharex=True)
#gint = (13990527,13854100)
#gint = (13871200,13854100)
g0 = 13901623 # atg pos common to all isoforms
for i in range(7):
    t = mamoisos[i]
    _drawone_tc2(axr[i], mamoccs[t], g0, pintsdic[t], t, mamoclrs[t])
    #_drawone_tc(axr[i], mamoccs[t], gint, pintsdic[t], t, mamoclrs[t])
fig.savefig(DIR+'mamo-domains-compressed.pdf')
```

```
RC 6235.0
RD 5996.0
RE 2424.0
RF 3097.0
RG 6102.0
RH 6235.0
RI 5996.0
```

In [9]:

```
fig, axr = P.subplots(8,1,figsize=(12,8*0.3))
#gint = (13990527,13854100)
gint = (13991527,13846200)
for i in range(7):
    t = mamoisos[i]
    #_drawone_tc2(axr[i], mamoccs[t], gint, pintsdic[t], t, mamoclrs[t])
    _drawone_tc(axr[i], mamoccs[t], gint, pintsdic[t], t, mamoclrs[t])
_drawscalebar(axr[7], mamoccs[t], gint, 10000, 1000)
fig.savefig(DIR+'mamo-domains-whole-nocompress-with-scalebar.pdf')
```

```
(-2696, 142631) 0 105978
(-5085, 140242) 0 117227
(-58184, 87143) 0 75327
(-44451, 100876) 0 77861
(-1000, 144327) 0 124666
(-2696, 142631) 0 137984
(-5085, 140242) 0 140327
(-5085, 140242) 0 140327
```

In [10]:

```
fig, axr = P.subplots(8,3,figsize=(12,8*0.3), sharey=True)

gint = (13856000,13853600)
print(N.abs(gint[0]-gint[1]))
for i in range(7):
    t = mamoisos[i]
    _drawone_tc(axr[i][2], mamoccs[t], gint, pintsdic[t], None, mamoclrs[t])
_drawscalebar(axr[7][2], mamoccs[t], gint, 0, 1000)

gint = (13871200,13869600)
print(N.abs(gint[0]-gint[1]))
for i in range(7):
    t = mamoisos[i]
    _drawone_tc(axr[i][1], mamoccs[t], gint, pintsdic[t], None, mamoclrs[t])
_drawscalebar(axr[7][1], mamoccs[t], gint, 0, 1000)

gint = (13885710,13882724)
print(N.abs(gint[0]-gint[1]))
for i in range(7):
    t = mamoisos[i]
    _drawone_tc(axr[i][0], mamoccs[t], gint, pintsdic[t], t, mamoclrs[t])
_drawscalebar(axr[7][0], mamoccs[t], gint, 0, 1000)

fig.savefig(DIR+'mamo-domains-zoomins-with-scalebar.pdf')
```

```
2400
(132831, 135231) 0 105978
(130442, 132842) 0 117227
(77343, 79743) 0 75327
(91076, 93476) 0 77861
(134527, 136927) 0 124666
(132831, 135231) 0 137984
(130442, 132842) 0 140327
(130442, 132842) 0 140327
1600
(117631, 119231) 0 105978
(115242, 116842) 0 117227
(62143, 63743) 0 75327
(75876, 77476) 0 77861
(119327, 120927) 0 124666
(117631, 119231) 0 137984
(115242, 116842) 0 140327
(115242, 116842) 0 140327
2986
(103121, 106107) 0 105978
(100732, 103718) 0 117227
(47633, 50619) 0 75327
(61366, 64352) 0 77861
(104817, 107803) 0 124666
(103121, 106107) 0 137984
(100732, 103718) 0 140327
(100732, 103718) 0 140327
```

In [ ]:

```

```
